# Unit costs and cost-effectiveness of a device to improve TB treatment adherence in China

**DOI:** 10.5588/ijtldopen.23.0451

**Published:** 2024-07-01

**Authors:** S. Sweeney, K. Fielding, X. Liu, J.A. Thompson, H. Dong, S. Jiang, Y. Zhao, S. Huan, A. Vassall

**Affiliations:** ^1^ London School of Hygiene & Tropical Medicine, London, UK;; ^2^ National Center for TB Control and Prevention, Chinese Center for Disease Control and Prevention, Beijing, China:; ^3^ PATH China Office, Beijing, China;; ^4^ Bill & Melinda Gates Foundation China Office, Beijing, China

**Keywords:** MERM, China, digital adherence technology, DAT, economics, tuberculosis, medication event reminder monitor

## Abstract

**BACKGROUND:**

Adherence to TB drugs is crucial for improving treatment outcomes. Digital adherence technologies can improve adherence; however, there is a lack of evidence on cost-effectiveness. This study aimed to explore the cost-effectiveness of medication event reminder monitors (MERM) in China compared with the standard of care, using results from a pragmatic, cluster-randomised superiority trial of an electronic MERM in China.

**METHODS:**

We collected primary unit cost data from the societal perspective, both at and above the health facility level. We estimated the incremental cost-effectiveness of MERM using a Markov model with a 20-year time horizon; a 3% discount rate was applied to costs and outcomes. We explored uncertainty through a series of sensitivity and scenario analyses.

**RESULTS:**

The incremental cost of MERM was $27.22 per patient. Probabilistic sensitivity analysis showed significant uncertainty about the intervention's cost-effectiveness. Changing assumptions around key parameters substantially affected our estimated incremental cost-effectiveness ratio.

**CONCLUSIONS:**

Although the incremental cost of the MERM box was low, current evidence does not indicate that the intervention would be cost-effective. However, the intervention's cost-effectiveness could improve if implemented as part of a broader strategy, including enhanced patient management.

## RÉSUMÉ

**CONTEXTE:**

Il est crucial de respecter les médicaments antituberculeux pour améliorer les résultats du traitement. Les technologies numériques peuvent améliorer l'observance, mais il existe un manque de preuves sur leur rapport coût-efficacité. Cette étude a examiné le rapport coût-efficacité des moniteurs de rappel d'événements médicamenteux (MERM, pour l’anglais, « *medication event reminder monitors* ») en Chine par rapport aux soins standards, en se basant sur les résultats d'un essai pragmatique randomisé en grappes d'un MERM électronique en Chine.

**MÉTHODES:**

Les coûts unitaires primaires du point de vue de la société ont été collectés et analysés à la fois au niveau de l'établissement de santé et au-delà. Pour évaluer le rapport coût-efficacité différentiel du MERM, nous avons utilisé un modèle de Markov sur une période de 20 ans, en appliquant un taux d'actualisation de 3% aux coûts et aux résultats. Afin de prendre en compte les incertitudes, nous avons effectué plusieurs analyses de sensibilité et de scénarios.

**RÉSULTATS:**

Le coût supplémentaire du MERM s'élevait à 27,22 $ par patient. L'analyse de sensibilité probabiliste a révélé une incertitude importante concernant le rapport coût-efficacité de l'intervention. La variation des hypothèses liées aux paramètres clés a eu un impact significatif sur le rapport coût-efficacité différentiel estimé.

**CONCLUSIONS:**

Bien que le coût différentiel de la boîte MERM soit faible, les données actuelles n'indiquent pas que l'intervention serait rentable. Toutefois, le rapport coût-efficacité de l'intervention pourrait être amélioré si elle était mise en œuvre dans le cadre d'une stratégie plus large, comprenant une meilleure prise en charge des patients.

Adherence to TB drugs is often cited as critical to treatment success and limiting the development of multidrug-resistant TB (MDR-TB).^1–3^ Many countries rely on directly observed therapy (DOT) to encourage adherence to anti-TB medications. There is also growing interest in using digital adherence technologies (DATs) as a more patient-centric alternative to DOT, which can be a burden on patient time and privacy.^4^

In 2017, the WHO recommended using DATs based on very low certainty of evidence.^5^ Early indications suggest that DATs could be cost-effective, but the evidence base is still evolving, complicating the application of existing evidence to policy decisions.^6–9^ Some studies have shown that medication reminders, combined with efforts to improve adherence, can significantly enhance treatment outcomes,^10–12^ suggesting that implementation strategies may influence impact.

This study aims to explore the potential cost-effectiveness of medication event reminder monitor (MERM) devices in China. We first present unit costs and cost-effectiveness of MERM devices as implemented in the trial. Using secondary evidence from the literature, we conduct a series of scenario analyses to explore our findings.

## METHODS

### Study population and settings

From January 2017 to March 2019, we conducted a pragmatic, cluster-randomised trial evaluating the superiority of an electronic medication event reminder monitor (MERM) over the standard of care (SOC) in 23 health facilities across four geographical areas of China.^13^ Study participants aged 18 years and older were enrolled at the initiation of treatment if they met the following criteria: they had pulmonary TB, tested positive on GeneXpert (Cepheid, Sunnyvale, CA, USA), had rifampicin-susceptible TB (RS-TB), were on a daily fixed-dose combination treatment regimen (2HRZE/4HR),^*^ and confirmed their ability to attend follow-up visits at 12 and 18 months post-treatment initiation to test for TB recurrence in cured patients. Participants provided written informed consent to participate in the trial. This study was approved by the Institutional Review Board of the Chinese Centers for Disease Control and Prevention (CDC), Beijing, China, and the Ethics Committee of the London School of Hygiene & Tropical Medicine, London, UK. The study design is described in detail in the main trial paper.^13^ Participant demographics are detailed in Supplementary Table S1.

### Intervention and comparators

All patients in both the intervention and SOC arms were provided with a MERM box to store their anti-TB drugs. MERM boxes provided daily audio and visual reminders to patients in the intervention arm; these reminders were muted for the SOC arm. Box opening was digitally recorded in both arms and considered a proxy for the dose taken. In the intervention arm, this information was available to the healthcare provider at routine monthly visits and could be used to inform decisions about further adherence support, such as increasing the frequency of home visits or phone calls or initiating DOT. Providers in the SOC arm could not see adherence data recorded by the MERM, and treatment management followed the SOC in each county; this could include self-administered treatment or DOT by healthcare workers and was determined by clinicians on a case-by-case basis.

### Model description

We developed a Markov model to represent the progression of a hypothetical cohort of 1,000 patients through different health states (i.e., active TB under treatment, active TB without treatment, post-TB/asymptomatic, death) and activities (i.e., first-line RS-TB treatment, RS-TB retreatment, rifampicin-resistant TB treatment, loss to follow-up [LTFU], etc.) under each treatment option over a time horizon of 20 years (Supplementary Figure S1). Patients were assumed to enter the model with active RS-TB at treatment initiation and progressed through the model at the end of each month. Initiation of retreatment after treatment failure or transference to MDR-TB treatment was assumed to be immediate as patients were already in care; treatment initiation after recurrence was assumed to follow national treatment initiation rates.^14^ We further assumed that a small proportion of patients with recurrence or treatment failure would develop acquired MDR-TB and would be initiated on standard treatment.^15^ The mean age at model entry was assumed to be 45 years, matching the trial population (Supplementary Table S2).

### Cost data collection and analysis

We collected primary unit cost data using a bottom-up, retrospective approach to resource use estimation. We estimated the full economic costs of MERM boxes from the societal perspective, reflecting the intervention's ‘real-world’ implementation, including start-up costs and above-service-level costs.

We estimated the above service-level costs in the Jiangxi, Jilin, and Zhejiang Provinces. Activities above the service level included MERM software development, distribution, training of physicians to use MERM, and supervision of sites by local CDC staff to support the use of MERM and any changes in patient management. Health facility-level costs, including staff time, building space, equipment, overheads, laboratory tests, and drugs, were estimated at nine health facilities (five interventions; four SOC). Activities at the facility level included outpatient visits, MERM check-in visits, inpatient bed days, home visits, phone calls, drug prescriptions, and laboratory tests.

Finally, we estimated the costs incurred by patients during their entire treatment episode by extracting detailed information on the quantity and prices paid by patients for outpatient visits, inpatient bed days, drugs and traditional Chinese medicines, and laboratory tests from patient records and financial systems for a random subsample of 20 patients per health facility (100 intervention; 80 SOC). The costs of traditional Chinese medicines were included because their prescription by health facilities as part of TB treatment is standard in China.^16–18^ First-line TB treatment is provided free to patients in China, along with a limited number of X-ray examinations and sputum smear tests; therefore, these costs were estimated as part of the facility-incurred cost. Patients are asked to pay out-of-pocket for other drugs or laboratory tests, including monitoring tests and traditional Chinese medicines.

We conducted a cluster-level analysis using the Stata package ‘*clan’* (Stata, College Station, TX, USA) to compare costs during the treatment episode between trial arms.^19^ A regression model was first run on each summary cost outcome to adjust for age, sex, occupation, migrant status, distance to clinic, education level, household expenditure, and smear result at treatment initiation, ignoring clustering and trial arm. Residuals were then summarised by cluster, and the cluster summaries were compared between the arms using linear regression.^20^ We observed significant cost reductions for one cost input in the intervention arm (discussed further in the Results section). The trial team could not identify a plausible causal pathway whereby this cost reduction could result from the intervention; therefore, we used the overall mean of all cost inputs in the parameterisation of the Markov model, assuming no real difference in costs between arms aside from the costs of MERM implementation.

Cost data were collected between April 2018 and August 2018. Costs were collected using standardised tools in Microsoft Excel and compiled using Stata v16. All costs were collected in Chinese yuan (CNY) and converted to United States dollars (US$) using the average mid-market exchange rate for the period of data collection (US$1 = CNY ¥6.50).^21^ Costs are presented in 2018 US$/CNY. All costs were discounted by 3%.^22^

### Treatment outcomes

We defined outcomes at the end of treatment as treatment completion or cure, treatment failure, death, switching to treatment for MDR-TB, and LTFU (Table 1). Risk ratios (RRs) and standard errors for treatment failure, death, and LTFU in the trial were calculated using a random effect log-linear regression model with a random effect for clusters. Only two patients switched to MDR-TB treatment; for this outcome, we assumed no difference between the arms and gave this a large standard error. A rate ratio was calculated by comparing arms using a cluster-level analysis: the log of the rate of recurrence was calculated for each cluster, and then these cluster-level summaries were compared between the arms using a *t*-test.

**Table 1.** Treatment outcome parameters for the model.

Treatment outcome	Outcomes in SOC trial arm (*n* = 1,300) *n* (%)	Outcomes in MERM trial arm (*n* =1,238) *n* (%)	Intervention vs. SOC arm Mean RR (95% CI)
Successful treatment outcome: completion or cure	1,097 (84.3)	1,050 (84.8)	
Poor treatment outcome: treatment failure	33 (2.5)	45 (3.6)	1.43 (0.88–2.32)
Poor treatment outcome: death	12 (0.9)	10 (0.8)	0.87 (0.36–2.14)
Poor treatment outcome: LTFU	156 (12.0)	133 (10.7)	0.90 (0.69–1.16)
Poor treatment outcome: switch to MDR-TB treatment	2 (0.2)	0	1.00 (0.02–64)
Recurrence in those with successful treatment	14	28	1.61 (0.75–3.45)

SOC = standard of care; MDR-TB = multidrug-resistant TB; SE = standard error; LTFU = loss to follow-up.

Outcomes for retreatment after recurrence or treatment failure and for MDR-TB treatment were based on national treatment outcomes (Supplementary Table S2).^23^

The monthly transition probabilities for all outcomes were calculated using the average treatment duration. Patient costs and outcomes were tallied over a time horizon of 20 years; this was adequate to capture all relevant costs and outcomes.

The measure of effectiveness for our incremental cost-effectiveness estimates was in terms of disability-adjusted life-years (DALYs) averted, chosen in discussion with the China CDC. We estimated the years of life lost using the weighted average life expectancy at death across the trial population. We estimated the years of life lived with a disability using disability weights for each health state sourced from the most recent Global Burden of Disease study^24^ (Table 2). Following evidence that there is a lifelong post-TB health burden even in patients with treatment success, we used a disability weight of 0.033 for a ‘post-TB’ state after cure or treatment completion^25^ and tested this in a scenario analysis. All DALYs were discounted at 3%.^22^

**Table 2.** Disability weights for all health states in the model.

Condition	DALY weight	SE	PSA distribution	Source
Post-TB	0.033	0.004	Beta	25
Active TB, no HIV	0.333	0.06	Beta	24
End of life	0.540	0.09	Beta	24
Death	1		Beta	24

DALY = disability-adjusted life-year; SE = standard error; PSA = probabilistic sensitivity analysis.

### Uncertainty and scenario analysis

We ran the primary analysis for 1,000 iterations, varying all parameters according to predefined distributions. Risk ratios for poor treatment outcomes varied according to log-normal distributions. Unit costs were varied using a gamma distribution, and all disability weights were varied following a beta distribution.^26^

We also conducted a series of scenario analyses to test the impact of uncertainty around specific key parameters and assumptions in our model. We first tested our hypothesis that observed differences in patient-incurred costs were irrelevant to the intervention by estimating cost-effectiveness using the adjusted mean difference for non-TB drug costs between the SOC and intervention groups, rather than the overall mean.

We next explored the possibility that the trial underestimated the long-term impact of improved adherence on recurrence. The overall percentage of relapses in the trial population was low (1.9% over 12 months), and the approach for documenting recurrence may not have been sufficiently sensitive. Therefore, we estimated cost-effectiveness using a standardised estimate of the risk of relapse in the year after treatment completion (2.8%) for patients in both arms with adherence lower than 90%.^27^ We applied a range of plausible risk ratios for recurrence in patients with adherence >90% in both arms (RR 0.34, 95% confidence interval [CI] 0.10–1.12).^6^

Finally, in the trial, although clinicians in the intervention had access to MERM data on drug adherence, patients did not report any change in patient management following the identification of non-adherence at monthly reviews. We investigated the idea that the intervention may have been more effective if more changes in patient management were initiated. In this scenario, we assumed that each patient with adherence <50% would receive daily home visits to reduce loss to follow-up (LTFU). We added costs equivalent to daily home visits and replaced the risk ratio for LTFU with a plausible range of reductions in LTFU from other studies on electronic medication monitor boxes (RR 0.59, 95% CI 0.43–0.80).^6^

We calculated the average incremental cost and the average disability-adjusted life years (DALYs) averted for each key parameter in our scenario analysis. We also varied these parameters over 1,000 iterations and estimated the percentage of model iterations where the intervention would be considered cost-effective under a national willingness-to-pay (WTP) threshold of 0.5 × gross domestic product per capita (US$5,072/¥32,967) per DALY averted.^9^

## RESULTS

### Unit cost estimates and cost analysis

Table 3 shows the total costs per patient episode in the intervention and SOC arms. Most cost differences between arms were not significant. There was a considerable, non-significant reduction in the costs of non-TB drugs (mean difference: –$24.01) and traditional Chinese medicines (mean difference: –$62.50) among patients in the intervention arm. However, we could not identify a pathway through which these differences could result from the intervention. The costs of MERM implementation are presented in further detail in Supplementary Tables S3–S7. Table 4 shows the total costs and outputs using the mean parameter estimates for the primary and scenario analyses. Patient-incurred costs were high in both arms (mean $433/¥2,818). The above-facility costs in implementing the MERM were $15.50 per patient episode (¥100.73).

**Table 3.** Total cost per patient episode by arm.*

	Mean SOC cost (US$)	Mean intervention cost (US$)	Adjusted mean difference (95% CI)^†^	Overall mean cost (95% CI) (US$)
Above-facility costs				
Software development		2.94	N/A^‡^	2.94
Training and supervision of HCWs		13.42	N/A^‡^	13.42
Distribution of equipment		2.80	N/A^‡^	2.80
Provider-incurred costs				
Inpatient bed days	72.19	78.85	15.94 (–78.83 to 110.72)	75.89 (54.54 to 97.24)
Phone calls	2.17	2.16	–0.05 (–0.63 to 0.53)	2.18 (2.12 to 2.25)
Home visits	2.29	1.65	–0.50 (–1.16 to 0.16)	1.90 (1.83 to 1.98)
Outpatient visits	100.29	92.59	–4.65 (–17.77 to 8.47)	96.01 (92.86 to 99.16)
First-line TB drug regimen	10.37	10.37	N/A^§^	10.37
MERM box	0	4.58	N/A^‡^	4.58
Patient-incurred costs				
Laboratory tests	152.75	144.64	1.96 (–84.33 to 88.24)	148.25 (136.26 to 160.24)
Indirect costs of patient time	36.18	40.45	11.83 (–2.65 to 26.32)	38.60 (31.28 to 45.92)
Patient transport costs	20.70	19.08	–0.90 (–3.49 to 1.69)	19.80 (19.16 to 20.43)
Non-TB drugs	90.49	41.48	–24.01 (–50.32 to 2.30)	63.27 (47.80 to 78.73)
Traditional Chinese medicines	217.38	139.86	–62.50 (–127.76 to 2.76)	174.31 (157.62 to 191.00)

* All cost estimates in 2018 US$; costs in 2018 CNY presented in Supplementary Table S4.

† Adjusted for annual household expenditure, sputum positivity, and residency within vs. outside the county of registration.

‡ Assumed costs of MERM were incurred only in intervention.

§ Assumed first-line TB drugs followed standard regimens in both SOC and intervention groups. SOC = standard of care; US$ = US dollars; CI = confidence interval; N/A = not available; HCW = healthcare worker; MERM = (electronic) medication event reminder monitor; CNY = Chinese yuan.

**Table 4.** Mean cost-effectiveness estimates.*

	Mean SOC costs (US$)	Mean intervention costs (US$)	Incremental costs/DALYs averted
Primary analysis			
Total costs	640.98	668.47	27.49
Above-facility level cost	0.00	15.50	15.50
Provider-incurred costs	208.42	218.42	9.99
Patient- incurred costs	432.56	434.56	2.00
Total DALYs	3.02	3.01	0.01
Incremental cost-effectiveness ratio			3,668.59
Scenario analysis: adjusted recurrence rates			
Total costs	641.95	665.22	23.27
Above-facility level cost	0.00	15.50	15.50
Provider-incurred costs	209.18	215.87	6.69
Patient- incurred costs	432.77	433.85	1.09
Total DALYs	3.03	2.96	0.07
Incremental cost-effectiveness ratio			344.35
Scenario analysis: adjusted patient costs			
Total costs	658.67	641.78	–16.89
Above-facility level cost	0.00	15.50	15.50
Provider-incurred costs	208.42	218.42	9.99
Patient- incurred costs	450.25	407.87	–42.38
Total DALYs	3.02	3.01	0.01
Incremental cost-effectiveness ratio			dominant
Scenario analysis: improved patient management			
Total costs	640.98	920.96	279.98
Above-facility level cost	0.00	134.65	134.65
Provider-incurred costs	208.42	345.98	137.56
Patient- incurred costs	432.56	440.34	7.78
Total DALYs	3.02	2.62	0.39
Incremental cost-effectiveness ratio			715.91

* All cost estimates in 2018 US$; estimates in 2018 CNY presented in Supplementary Table S8.

SOC = standard of care; US$ = US dollars; DALY = disability-adjusted life-year; CNY = Chinese yuan.

### Cost-effectiveness

In our primary analysis, the intervention arm averted 0.01 DALYs at an incremental cost of $27 (¥176), making the intervention more expensive and slightly more effective than the SOC. Over half (57%) of the incremental costs were incurred above the facility level. The mean incremental cost-effectiveness ratio (ICER) was $2,412 (¥15,680), falling well below the willingness-to-pay threshold of $5,072 (¥32,967) per DALY averted. Incremental costs for other outcomes are listed in Supplementary Table S11.

Our cost-effectiveness results were highly uncertain. The results of the probabilistic sensitivity analyses are shown in the Figure and Supplementary Table S6. Only 39% of the model iterations in the primary analysis fell below the WTP. In 48% of the model iterations, the intervention was more costly and less effective than the SOC.

**Figure.**
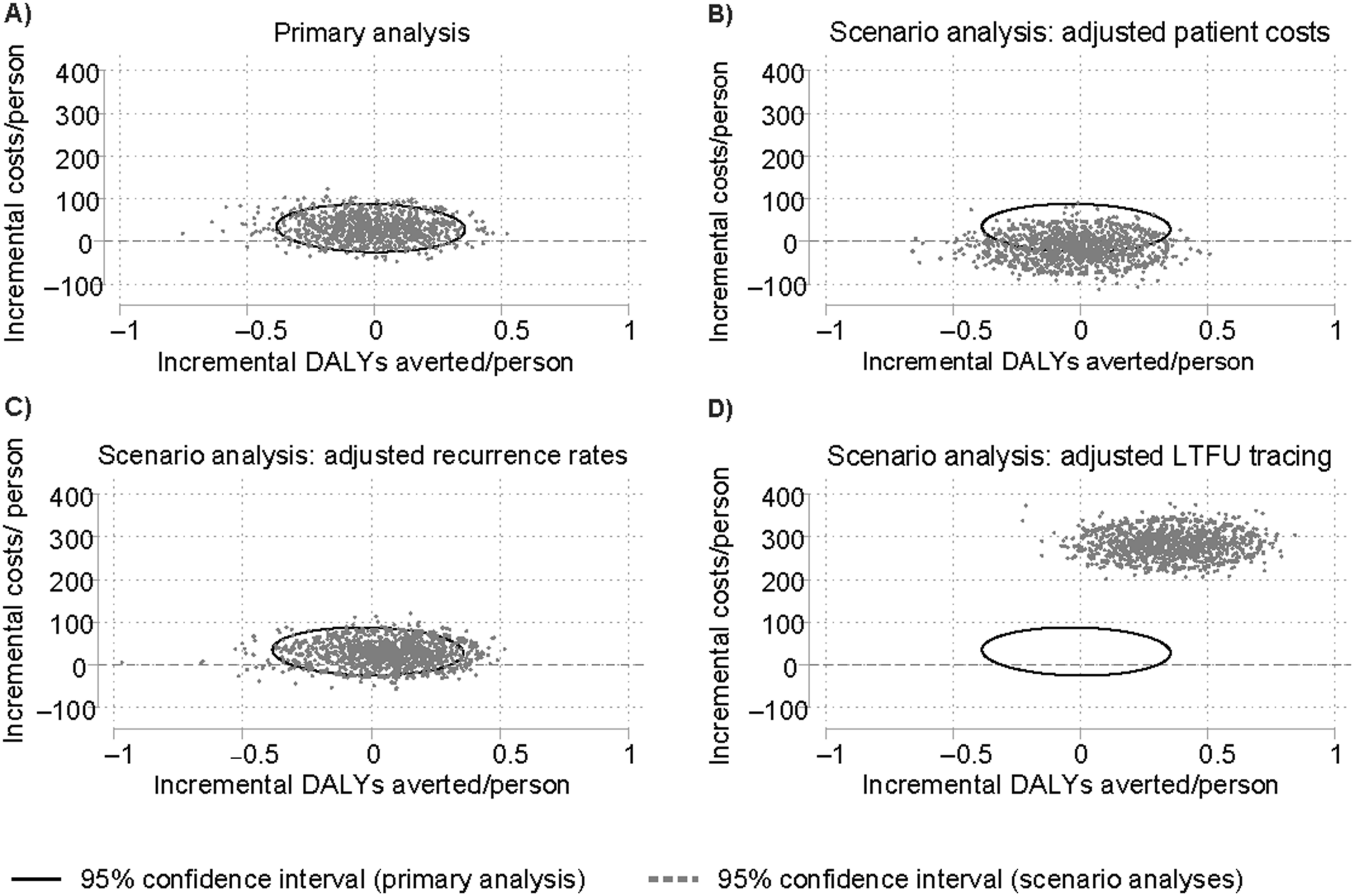
Probabilistic sensitivity analysis results. All cost estimates in 2018 US dollars. **A)** Results of the primary analysis varying all parameters simultaneously over 1,000 iterations. **B)** Results of the scenario analysis using the adjusted mean difference for costs for non-TB drugs between the SOC and intervention groups. **C)** Results of the scenario analysis applying a range of plausible risk ratios for recurrence in patients with adherence >90% in both arms. **D)** Results of the scenario analysis adding added costs equivalent to daily home visits for those with adherence <50% and replacing the risk ratio for LTFU with a plausible range of reductions in LTFU for directly observed therapy. DALY = disability-adjusted life-years; LTFU = loss to follow-up; SOC = standard of care.

Changing our assumptions about the patient-incurred costs for non-TB drugs reduced costs in the intervention compared with the SOC. Adjusting recurrence rates improved the mean cost-effectiveness of the intervention. In our scenario analysis, assuming improved management for those with poor adherence to prevent loss to follow-up (LTFU), 93% of model iterations were cost-effective. A threshold analysis of this scenario indicated that additional monthly LTFU prevention costs of up to $105 (¥682) per month would likely be cost-effective (Supplementary Figure S2). Ignoring post-TB disability in our DALY calculations had no impact on the incremental DALYs averted (Supplementary Table S9).

## DISCUSSION

Our mean estimate of the incremental cost-effectiveness of MERM was $2,412 (¥15,680) per DALY averted, which is cost-effective at a threshold of 0.5x GDP per capita. Our analysis is limited by the small sample size for costing, which makes it challenging to identify any practical difference in cost between arms. Our analysis also showed extreme uncertainty surrounding the cost-effectiveness of the intervention and which quadrant of the cost-effectiveness plane the intervention fell into. In our primary analysis, nearly half of the model iterations showed that the intervention was more costly and less effective than the SOC. Therefore, we cannot conclude that MERM boxes are cost-effective.

The driver of uncertainty in our results is uncertainty around the impact of MERM boxes on health outcomes. While our trial found no evidence that MERM improved treatment outcomes, trials in Uganda, Peru, and Tibet showed that medication reminders improved outcomes. In these studies, monitors were supplemented alongside efforts to encourage adherence, including automated text message reminders, contacting family members, individual follow-up, motivational messaging, support from TB survivors, and a gamification component. Although there is no current cost-effectiveness evidence from these studies, forthcoming estimates may add to the evidence base on the economics of MERMs.

Most costs in both arms were incurred by patients who reported high expenditures on laboratory tests, traditional Chinese medicines, and non-TB-related drugs. Our cost data showed a large reduction in patient-incurred costs for non-TB drugs, although it is unclear whether this resulted from the intervention. High patient costs for TB treatment in China are often cited as causes of impoverishment and barriers to care. Further investigation of the impacts of different approaches to patient management on patient-incurred costs would be a useful start to identify potential avenues for reducing patient-incurred costs.

Improving adherence is a priority of the Chinese National TB Programme. Given the very low cost of MERM ($27; ¥177) and the high potential to improve patient adherence, the results of this study have improved confidence within the Chinese National TB Programme regarding the value of investing in digital tools for TB management. China is currently working to expand the use of digital tools for TB management, including MERM devices.

## Supplementary Material



## Footnotes

* H = isoniazid; R = rifampicin; Z = pyrazinamide; E = ethambutol. Numbers before the letters indicate the duration in months of the phase of treatment.
