# Supplementary material for: Unit costs and cost-effectiveness of a device to improve TB treatment adherence in China
Source: IJTLD Open. 2024 Jul 1;1(7):299–305. doi: 10.5588/ijtldopen.23.0451 (PMC11257087; doi:10.5588/ijtldopen.23.0451)
Supplement: Supplementary file 1 [file ijtldopen0451_supplementarydata1.docx]

###### <http://dx.doi.org/10.5588/ijtldopen.23.0451>

# **Unit costs and cost-effectiveness of a device to improve TB treatment adherence in China**

###### **Supplementary Table S1** Patient characteristics

|  | **Patient costing dataset** | | | | **Full patient costing dataset (n=180), n (%)** | | **Full trial dataset**  **(n = 3,075), n (%)** | |
| --- | --- | --- | --- | --- | --- | --- | --- | --- |
|  | **SOC (n=80), n (%)** | | **Intervention (n=100), n (%)** | |  |  |  |  |
| Hospitalized (n, %) | 24 | 30% | 27 | 27% | 51 | 28% |  |  |
| Married (n, %) | 60 | 75% | 77 | 77% | 137 | 76% | 2275 | 74% |
| Distance to clinic, km (mean) | 22.2 |  | 18.5 |  | 19.2 |  | 19.3 |  |
| Education (n, %) |  |  |  |  |  |  |  |  |
| Illiterate | 12 | 15% | 7 | 7% | 19 | 11% | 250 | 8% |
| Primary School | 20 | 25% | 32 | 32% | 52 | 29% | 879 | 29% |
| Junior Middle School | 25 | 31% | 35 | 35% | 60 | 33% | 1144 | 37% |
| High school or above | 23 | 29% | 26 | 26% | 49 | 27% | 802 | 26% |
| Medical insurance |  |  |  |  |  |  |  |  |
| New Rural Cooperative Medical System | 50 | 63% | 71 | 71% | 121 | 67% | 1,787 | 58% |
| Urban Residents’ Medical Insurance | 9 | 11% | 8 | 8% | 17 | 9% | 432 | 14% |
| Medical Insurance for Urban Workers | 10 | 13% | 11 | 11% | 21 | 12% | 313 | 10% |
| Government employee Medical Service | 0 | 0% | 0 | 0% | 0 | 0% | 9 | 0% |
| Commercial Insurance | 1 | 1% | 1 | 1% | 2 | 1% | 8 | 0% |
| No Insurance | 10 | 13% | 9 | 9% | 19 | 11% | 526 | 17% |
| Household size (n, %) |  |  |  |  |  |  |  |  |
| under 4 members | 36 | 45% | 52 | 52% | 88 | 49% | 1223 | 40% |
| 4-8 members | 40 | 50% | 45 | 45% | 85 | 47% | 1719 | 56% |
| over 8 members | 4 | 5% | 3 | 3% | 7 | 4% | 133 | 4% |
| Age Group (n, %) |  |  |  |  |  |  |  |  |
| under 40 years | 31 | 39% | 47 | 47% | 78 | 43% | 1333 | 43% |
| 40-60 years | 30 | 38% | 24 | 24% | 54 | 30% | 995 | 32% |
| over 60 years | 19 | 24% | 29 | 29% | 48 | 27% | 747 | 24% |
| Employment status (n, %) |  |  |  |  |  |  |  |  |
| Unemployed | 12 | 15% | 13 | 13% | 25 | 14% | 331 | 11% |
| Employee | 18 | 23% | 28 | 28% | 46 | 26% | 975 | 32% |
| Farmer | 43 | 54% | 49 | 49% | 92 | 51% | 1564 | 51% |
| Retiree/Student | 7 | 9% | 6 | 6% | 13 | 7% | 185 | 6% |
| Monthly Household Expenditure (n, %) |  |  |  |  |  |  |  |  |
| less than 1,000 CNY | 2 | 3% | 7 | 7% | 9 | 5% | 289 | 9% |
| 1,000-3,000 CNY | 43 | 54% | 54 | 54% | 97 | 54% | 1565 | 51% |
| more than 3,000 CNY | 35 | 44% | 39 | 39% | 74 | 41% | 1221 | 40% |
| Sputum result |  |  |  |  |  |  |  |  |
| Sputum positive | 42 | 53% | 45 | 45% | 87 | 48% | 1149 | 31% |
| Sputum negative | 38 | 48% | 55 | 55% | 93 | 52% | 1926 | 52% |

SOC standard of care, CNY Chinese Yuan, km killometers

###### **Supplementary Table S2** Model parameters

| **Parameter** | **Value** | **SE** | **PSA Distribution** | **Reference** |
| --- | --- | --- | --- | --- |
| Average age at model start (years) | 45 |  | N/A | assumption |
| Average body weight (kilograms) | 51-70 |  | N/A | assumption |
| DS-TB retreatment cohort outcomes: success / died / treatment failed / LTFU | 85.4% / 3.5% / 4.8% / 6.4% |  | N/A | (World Health Organization, 2021) |
| DR-TB retreatment cohort outcomes: success / died / treatment failed / LTFU | 55.1% / 5.5% / 7.0% / 32.5% |  | N/A | (World Health Organization, 2021) |
| Discount rates for costs and effects | 3% |  | N/A | (Wilkinson *et al.*, 2016) |
| Annual risk of relapse: year 1 / year 2 / year 3 / year 4 post-treatment | 2.8% / 1.0% / 0.4% / 0.3% | 0.4% | Normal | (Marx *et al.*, 2014) |
| Hazard ratio for relapse among people with HIV | 2.40 |  | N/A | (Naidoo and Dookie, 2018) |
| Annual likelihood of return to care after LTFU | 28% | 3% | Normal | (Marx *et al.*, 2012) |
| Annual likelihood of return to care after relapse | 87% |  | N/A | (World Health Organization, 2021) |
| Access to end of life care | 25% | 5% | Normal | assumption |
| Monthly probability of death for end-of-life care | 6.86% | 0.69% | Beta | (Franke *et al.*, no date) |
| Monthly probability of death following LTFU | 6.86% | 0.69% | Beta | (Franke *et al.*, no date) |

SE standard error; PSA probabilistic sensitivity analysis; N/A not applicable; HIV human immunodeficiency virus; LTFU lost to follow-up

###### **Supplementary Table S3** Total costs per patient episode, by arm, CNY

|  | **SOC mean cost (CNY)** | **Intervention mean cost (CNY)** | **Adjusted mean difference^1^**  **(95% CI)** | **p value** | **Overall mean cost (CNY)**  **(95% CI)** |
| --- | --- | --- | --- | --- | --- |
| **Above-facility costs** |  |  |  |  |  |
| Software development |  | 19.12 | n/a* |  | 19.12 |
| Training & supervision of HCW |  | 87.21 | n/a* |  | 87.21 |
| Distribution of equipment |  | 18.19 | n/a* |  | 18.19 |
| **Provider-incurred costs** |  |  |  |  |  |
| Inpatient bed days | 469.23 | 512.54 | 42.90 (-711.77 to 797.57) | 0.90 | 493.29 (354.54 to 632.04) |
| Phone calls | 14.58 | 13.78 | -0.50 (-4.54 to 3.54) | 0.80 | 14.19 (13.77 to 14.60) |
| Home visits | 14.62 | 10.08 | -3.77 (-8.49 to 0.96) | 0.11 | 12.38 (11.90 to 12.86) |
| Outpatient visits | 651.89 | 601.84 | -43.25 (-160.97 to 74.47) | 0.41 | 624.09 (603.60 to 644.57) |
| First-line TB drug regimen | 67.40 | 67.40 | n/a3 |  | 67.40 |
| MERM box | 0 | 29.77 | n/a* |  | n/a* |
| **Patient-incurred costs** |  |  |  |  |  |
| Lab tests | 992.88 | 940.19 | -33.84 (-691.41 to 623.72) | 5.89 | 963.60 (885.67 to 1041.54) |
| Indirect costs of patient time | 234.59 | 263.92 | 53.59 (-151.78 to 258.96) | 3.62 | 250.88 (203.31 to 298.45) |
| Patient transport costs | 134.52 | 124.02 | -8.97 (-31.73 to 13.78) | 2.48 | 128.69 (124.57 to 132.81) |
| Non-TB drugs | 588.21 | 269.65 | -303.67 (-530.97 to -76.38) | 0.10 | 411.23 (310.73 to 511.74) |
| Traditional Chinese medicines | 1412.94 | 909.08 | -500.08 (-1016.70 to 16.53) | 0.36 | 1133.02 (1024.54 to 1241.49) |

HCW health care workers; CI confidence interval; MERM electronic medication event reminder monitor. All cost estimates in 2018 Chinese Yuan (CNY). ^1^ adjusted for annual household expenditure, sputum positivity, and residency within vs. outside the county of registration . ^2^ assumed costs of MERM were incurred only in intervention. ^3^ assumed first-line TB drugs followed standard regimens in both SOC and intervention groups

###### **Supplementary Table S4** Annualized costs of MERM implementation, above facility level, USD

|  | **Jiangxi Province** | **Jilin Province** | **Zhejiang Province** |
| --- | --- | --- | --- |
| **Number of health facilities in SOC arm** | 4 | 2 | 6 |
| **Number of health facilities in intervention arm** | 3 | 2 | 6 |
| **Mean above-facility costs, per health facility** |  |  |  |
| Software development^1^ | 404.29 | 404.29 | 404.29 |
| Training & supervision of health care workers^2^ | 815.35 | 2,294.28 | 2,092.70 |
| Delivery & distribution of equipment^2^ | 100.30 | 363.64 | 508.25 |
| **Mean above-facility costs, per patient** |  |  |  |
| Software development^1^ | 2.70 | 3.62 | 3.11 |
| Training & supervision of health care workers^2^ | 5.53 | 20.38 | 16.10 |
| Delivery & distribution of equipment^2^ | 0.67 | 3.26 | 3.91 |

^1^ annualized, useful life expectancy 10 years; ^2^ annualized, useful life expectancy 3 years. All cost estimates in 2018 US Dollars (USD)

###### **Supplementary Table S5** Annualized costs of MERM implementation, above facility level, CNY

|  | **Jiangxi Province** | **Jilin Province** | **Zhejiang Province** |
| --- | --- | --- | --- |
| **Number of health facilities in SOC arm** | 4 | 2 | 6 |
| **Number of health facilities in intervention arm** | 3 | 2 | 6 |
| **Mean above-facility costs, per health facility** |  |  |  |
| Software development^1^ | 2,627.88 | 2,627.88 | 2,627.88 |
| Training & supervision of health care workers^2^ | 5,299.77 | 14,912.82 | 13,602.55 |
| Delivery & distribution of equipment^2^ | 651.95 | 2,363.66 | 3,303.62 |
| **Mean above-facility costs, per patient** |  |  |  |
| Software development^1^ | 17.55 | 23.53 | 20.21 |
| Training & supervision of health care workers^2^ | 35.94 | 132.47 | 104.65 |
| Delivery & distribution of equipment^2^ | 4.35 | 21.19 | 25.41 |

^1^ annualized, useful life expectancy 10 years; ^2^ annualized, useful life expectancy 3 years. All cost estimates in 2018 Chinese Yuan (CNY)

###### **Supplementary Table S6** Mean provider costs of MERM implementation, at the facility level, USD

|  | **Jiangxi Province**  **Mean (95% CI)** | **Jilin Province**  **Mean (95% CI)** | **Zhejiang Province**  **Mean (95% CI)** |
| --- | --- | --- | --- |
| **Mean staff time per outpatient visit (minutes)** |  |  |  |
| Intervention | 13.3 (5.4, 21.1) | 11.8 (9.2, 14.4) | 5.5 (4.4, 6.7) |
| Comparator | 8.3 (4.9, 11.7) | 7.7 (5.5, 9.9) | 7.2 (4.8, 9.6) |
| **Mean staff cost per outpatient visit** |  |  |  |
| Intervention | 1.2 (0.5, 1.9) | 0.9 (0.7, 1.2) | 0.4 (0.3, 0.4) |
| Comparator | 0.7 (0.4, 1.0) | 0.7 (0.5, 0.9) | 1.0 (0.5, 1.6) |
| **Mean cost per home visit** |  |  |  |
| Staff time costs | 1.8 (1.8, 1.8) | 1.7 (1.7, 1.8) | 1.9 (1.5, 2.3) |
| Transport costs | 2.25 | 2.25 | 2.25 |
| Estimated costs for MERM box |  |  |  |
| Total price per box | 12.97 | 12.97 | 12.97 |
| Annualized cost per year^1^ | 4.58 | 4.58 | 4.58 |

^1^ useful life expectancy 3 years. CI confidence interval. All cost estimates in 2018 US Dollars (USD).

###### **Supplementary Table S7** Mean provider costs of MERM implementation, at the facility level, CNY

|  | **Jiangxi Province**  **Mean (95% CI)** | **Jilin Province**  **Mean (95% CI)** | **Zhejiang Province**  **Mean (95% CI)** |
| --- | --- | --- | --- |
| **Mean staff time per outpatient visit (minutes)** |  |  |  |
| Intervention | 13.3 (5.4, 21.1) | 11.8 (9.2, 14.4) | 5.5 (4.4, 6.7) |
| Comparator | 8.3 (4.9, 11.7) | 7.7 (5.5, 9.9) | 7.2 (4.8, 9.6) |
| **Mean staff cost per outpatient visit** |  |  |  |
| Intervention | 7.80 (3.25 - 12.35) | 5.85 (4.55 - 7.80) | 2.60 (1.95 - 2.60) |
| Comparator | 4.55 (2.60 - 6.50) | 4.55 (3.25 - 5.85) | 6.50 (3.25 - 10.40) |
| **Mean cost per home visit** |  |  |  |
| Staff time costs | 11.70 (11.70 - 11.70) | 11.05 (11.05 - 11.70) | 12.35 (9.75 - 14.95) |
| Transport costs | 14.62 | 14.62 | 14.62 |
| Estimated costs for MERM box |  |  |  |
| Total price per box | 84.30 | 84.30 | 84.30 |
| Annualized cost per year^1^ | 29.77 | 29.77 | 29.77 |

^1^ useful life expectancy 3 years. CI confidence interval. All cost estimates in 2018 Chinese Yuan (CNY).

###### **Supplementary Table S8** Mean cost-effectiveness estimates, CNY

|  | **Mean SOC costs (CNY)** | **Mean Intervention costs (CNY)** | **Incremental costs / DALYs averted** |
| --- | --- | --- | --- |
| **Primary analysis** |  |  |  |
| Total costs | ¥4,166.37 | ¥4,343.33 | ¥176.95 |
| *Above-facility level cost* | ¥0.00 | ¥100.73 | ¥100.73 |
| *Provider-incurred costs* | ¥1,171.85 | ¥1,210.07 | ¥38.22 |
| *Patient- incurred costs* | ¥2,811.63 | ¥2,824.25 | ¥12.62 |
| Total DALYs | 3.02 | 3.00 | 0.01 |
| ICER |  |  | ¥15,680.42 |
| **Scenario analysis: adjusted recurrence rates** |  |  |  |
| Total costs | ¥4,172.65 | ¥4,323.93 | ¥151.28 |
| *Above-facility level cost* | ¥0.00 | ¥100.73 | ¥100.73 |
| *Provider-incurred costs* | ¥1,174.43 | ¥1,202.08 | ¥27.65 |
| *Patient- incurred costs* | ¥2,812.99 | ¥2,820.04 | ¥7.06 |
| Total DALYs | 3.03 | 2.96 | 0.07 |
| ICER |  |  | ¥2,238.25 |
| **Scenario analysis: adjusted patient costs** |  |  |  |
| Total costs | ¥4,250.57 | ¥4,205.18 | -¥45.39 |
| *Above-facility level cost* | ¥0.00 | ¥100.73 | ¥100.73 |
| *Provider-incurred costs* | ¥1,171.85 | ¥1,210.07 | ¥38.22 |
| *Patient- incurred costs* | ¥2,895.83 | ¥2,686.10 | -¥209.72 |
| Total DALYs | 3.02 | 3.00 | 0.01 |
| ICER |  |  | dominant |
| **Scenario analysis: improved management** |  |  |  |
| Total costs | ¥4,166.37 | ¥5,939.54 | ¥1,773.17 |
| *Above-facility level cost* | ¥0.00 | ¥868.92 | ¥868.92 |
| *Provider-incurred costs* | ¥1,171.85 | ¥2,023.54 | ¥851.69 |
| *Patient- incurred costs* | ¥2,811.63 | ¥2,839.70 | ¥28.07 |
| Total DALYs | 3.02 | 2.85 | 0.17 |
| ICER |  |  | ¥10,488.58 |

SOC standard of care. DALYs disability adjusted life years. ICER incremental cost-effectiveness ratio. All cost estimates in 2018 Chinese Yuan (CNY)

###### **Supplementary Table S9** Scenario analysis with post-TB disability ignored, USD

|  | Control | Intervention | Incremental costs /  DALYs averted |
| --- | --- | --- | --- |
| Total costs | $640.98 | $668.20 | $27.22 |
| Above-facility level cost | $0.00 | $15.50 | $15.50 |
| Provider-incurred costs | $208.42 | $218.21 | $9.78 |
| Patient- incurred costs | $432.56 | $434.50 | $1.94 |
| Total DALYs | 2.60 | 2.59 | 0.01 |
| ICER |  |  | $2,281.02 |

###### **Supplementary Table S10** Scenario analysis with post-TB disability ignored, CNY

|  | Control | Intervention | Incremental costs /  DALYs averted |
| --- | --- | --- | --- |
| Total costs | ¥4,166.37 | ¥4,343.33 | ¥176.95 |
| Above-facility level cost | ¥0.00 | ¥100.73 | ¥100.73 |
| Provider-incurred costs | ¥1,354.75 | ¥1,418.35 | ¥63.60 |
| Patient- incurred costs | ¥2,811.63 | ¥2,824.25 | ¥12.62 |
| Total DALYs | 2.60 | 2.59 | 0.01 |
| ICER |  |  | ¥14,826.62 |

###### **Supplementary Table S11** Incremental cost per empiric / clinical outcome

|  | **Outcomes** | | **Incremental cost per outcome** |
| --- | --- | --- | --- |
| **Outcomes** | **Control** | **Intervention** |  |
| **Treatment outcomes** |  |  |  |
| Composite successful treatment outcome at 6 months | 239/1388 (16%) | 224/1298 (16%) | $293.42 |
| Conversion to negative smear after 2 months | 689/759 (89%) | 639/729 (89%) | dominated |
| **Adherence** |  |  |  |
| Patient that never missed 2 or more consecutive doses | 157/1306 (12%) | 391/1261 (31%) | $144.68 |
| Patient that never missed 3 or more consecutive doses | 319/1306 (24%) | 790/1261 (63%) | $70.49 |
| Patient that never missed 4 or more consecutive doses | 478/1306 (37%) | 965/1261 (77%) | $68.73 |

###### **Supplementary Table S12** Full probabilistic sensitivity analysis results

| **Quadrant / Cost-effectiveness** | **Primary Analysis** | **Scenario analysis: adjusted patient costs** | **Scenario analysis: adjusted recurrence rates** | **Scenario analysis: adjusted LTFU tracing** |
| --- | --- | --- | --- | --- |
| Quadrant 1: Cost-effective | 39% | 12% | 55% | 93% |
| Quadrant 1: Not cost-effective | 2% | 1% | 2% | 5% |
| Quadrant 2: Cost-saving | 5% | 34% | 10% | 0% |
| Quadrant 3: Detrimentally cost-effective | 6% | 35% | 5% | 0% |
| Quadrant 3: Not cost-effective | 0% | 1% | 0% | 0% |
| Quadrant 4: Dominated | 48% | 16% | 28% | 2% |

###### **Supplementary Figure S1** Markov model diagram


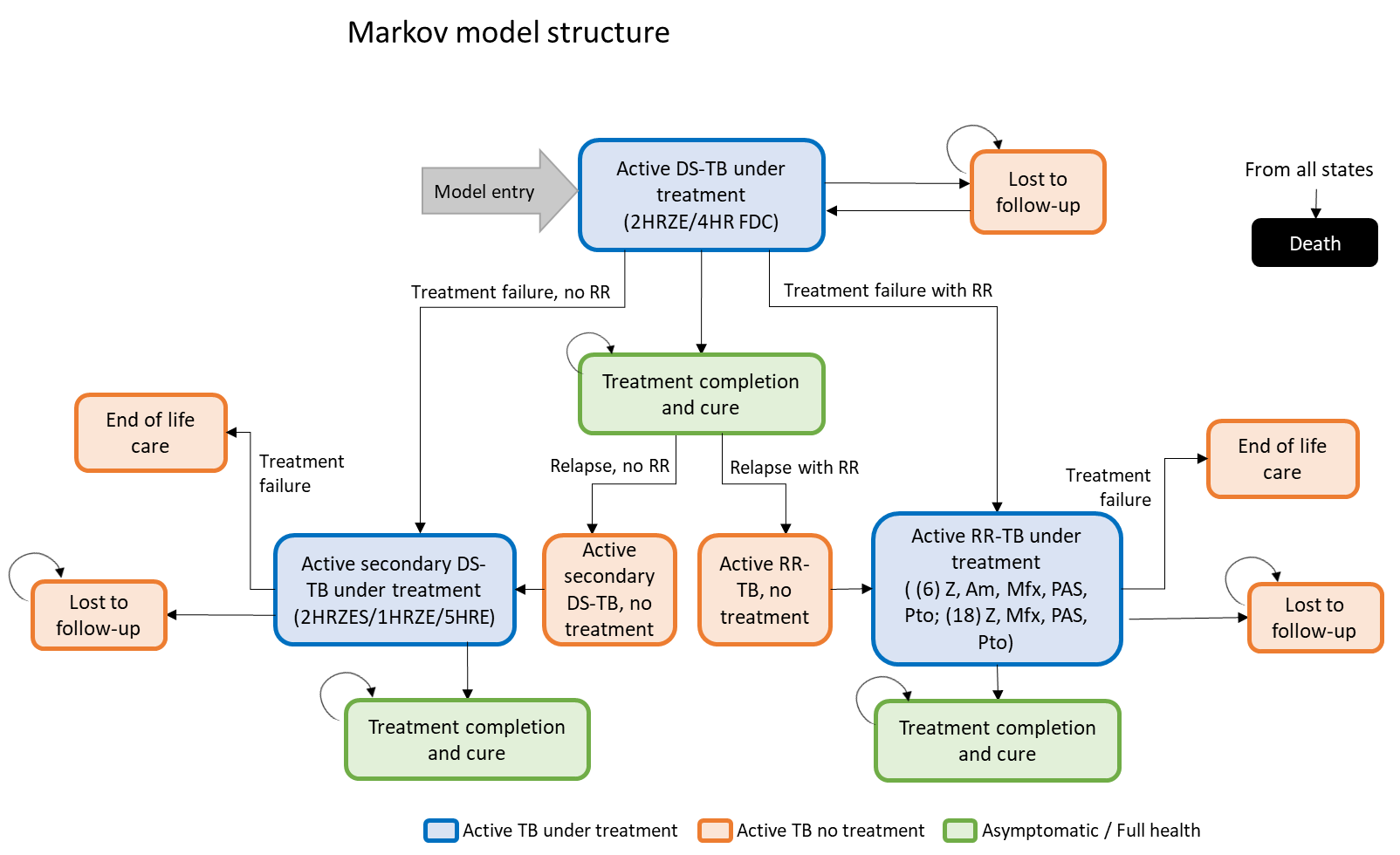


##### **Supplementary Figure S2** Threshold analysis for LTFU tracing


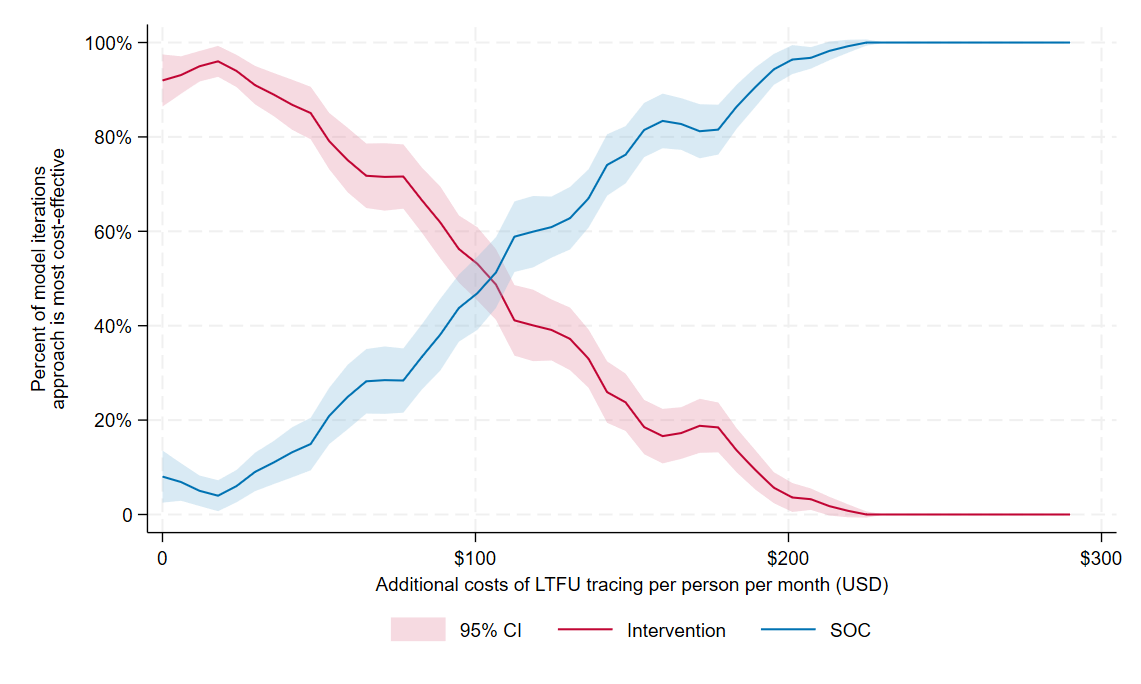


# **Supplementary Data**: Cost data collection process

## Above service-level unit cost collection

Above service-level costs were estimated using a top-down approach in nine health facilities (5 intervention; 4 SOC). The number of facilities included in primary cost data collection was driven by logistics and access to the health facilities. Resources spent before and after the start of patient enrolment were identified through retrospective review of China CDC administrative records, and through interviews and observations with local CDC staff in each province. Costs incurred at the Central level by China CDC, and at the Provincial level, were allocated per health facility using step-down accounting. As both comparator and intervention facilities received MERM boxes along with training and supervision, we allocated shared costs such as software development equally across all health facilities. Research costs were excluded from the cost analysis.

Above-facility costs of MERM distribution were estimated separately in comparator and intervention health facilities according to the number of MERM devices distributed to each facility. Province-level costs of trainings and supervisions were allocated equally across participating facilities. Costs incurred at the central level by the China CDC for MERM software development and coordination with local CDC offices were allocated equally across all participating health facilities. All above-facility costs were annualized with the conservative assumption that MERM software would have a useful life expectancy of 10 years, and that MERM boxes and corresponding training/supervision on their implementation would have useful life expectancies of 3 years. A discount rate of 3% was used for annualization of economic costs, and financial costs were annualized using straight-line depreciation.

## Service level unit cost collection

Facility-level costs were estimated using a bottom-up ingredients approach. We estimated the total staff time spent per outpatient visit, including use of the MERM, through observation and interviews with health facility staff. We estimated the provider costs of staff time per outpatient visit as the average number of minutes per consultation and the salary per minute for the observed clinician. Average staff salaries were sourced from CDC to estimate the cost of provider time per minute. Other provider-incurred costs for outpatient visits, including the costs of the clinic building, equipment, other administrative costs, and other non-personnel expenditures, were sourced from the literature due to a lack of primary data (Fitzpatrick *et al.*, 2015). Provider-incurred costs of home visits and phone calls were estimated based on expert opinion and existing literature; this included costs of staff time and transport costs (Zhao *et al.*, 2013).

Patient costs included direct medical, direct non-medical, and indirect costs. Direct medical costs included consultation fees, drug fees, and fees for laboratory tests. We estimated costs incurred by patients during their entire treatment period through extraction of detailed information on the quantity and prices paid by patients for outpatient visits, inpatient bed-days, drugs, and laboratory tests from patient records and financial systems. This was done for a random sub-sample of 20 patients per health facility, in nine facilities (4 facilities in the SoC arm, and 5 in the intervention arm). Characteristics for patients participating in the patient costing exercise are presented in Supplementary Table S1.

We estimated indirect patient costs as a function of individual income per hour, multiplied by the number of hours spent in consultation. We collected total time spent at the health facility for each outpatient clinic visit through observation, tracking patients from the time of their first consultation on a given day, until the end of their last consultation on the same day. Total patient time per visit therefore included time seeing multiple providers, waiting time, and time for laboratory tests. We estimated travel time as a function of the distance from patients’ house to the health facility where they received treatment, assuming a mean travel time of 2.8 minutes per kilometre (Engelfriet and Koomen, 2018), and assumed patients encountered a mean transport cost of 15 Yuan per round trip (Zhao *et al.*, 2013). We were unable to account for other direct non-medical costs, such as accommodation, due to a lack of data.

We collected detailed information on all drugs, including traditional Chinese medicines, and ‘other’ non-TB related drugs that patients purchased through retrospective review of medical records. We estimated the costs incurred by of. Other drugs included vitamins, supplements, and drugs to treat side-effects of TB treatment. Some participant records showed out-of-pocket payment for anti-TB drugs; to avoid double counting these drugs costs from the societal perspective we assumed all anti-TB drugs costs were incurred by the provider, and followed standard treatment regimens. Following WHO treatment guidelines, first-line treatment was assumed be the standard 6-month HRZE fixed-dose regimen (World Health Organization, 2022). Following treatment failure or relapse, we assumed patients would be retreated with a secondary regimen of 2HRZES/1HRZE/5HRE (World Health Organization, 2022). Patients with acquired RR-TB were assumed to follow the standard 18-month RR-TB treatment regimen used in China ((6) Z, Am, Mfx, PAS, Pto; (12) Z, Mfx, PAS, Pto) (Fitzpatrick *et al.*, 2015).

Insurance reimbursements were estimated according to the insurance scheme that patients were subscribed to. We assumed average reimbursement rates for inpatient care was 70.26% for patients enrolled in the Medical Insurance for Urban Workers scheme, 50.98% for those in the Urban Residents’ Medical Insurance and 48% for those in the New Rural Cooperative Medical System (Xiong *et al.*, 2018). Evidence has shown that reimbursements for outpatient services, particularly at TB dispensaries, are much lower than reimbursements for inpatient services, in some cases as low as 5% of out-of-pocket expenses (Xiang *et al.*, 2016). We conservatively assumed a mean real reimbursement rate of 20% out-of-pocket payments for outpatient services for all insurance schemes (Xiang *et al.*, 2016).

## References

Engelfriet, L. and Koomen, E. (2018) The impact of urban form on commuting in large Chinese cities, *Transportation*, 45 (5), pp. 1269–1295. DOI:10.1007/s11116-017-9762-6.

Fitzpatrick, C., Hui, Z., Lixia, W., Renzhong, L., Yunzhou, R., Mingting, C., *et al.* (2015) Cost–effectiveness of a comprehensive programme for drug-resistant tuberculosis in china, *Bulletin of the World Health Organization*, 93 (11), pp. 775–784. DOI:10.2471/BLT.14.146274.

Franke, M. F., Appleton, S. C., Bayona, J., Arteaga, F., Palacios, E., Llaro, K., *et al.* [no date] Risk Factors and Mortality Associated with Default from Multidrug-Resistant Tuberculosis Treatment. DOI:10.1086/588292.

Institute for Health Metrics and Evaluation (2019) Global Burden of Disease Study Disability Weights. Available from: https://ghdx.healthdata.org/record/ihme-data/gbd-2019-disability-weights [Accessed 17 November 2023].

Marx, F. M., Dunbar, R., Enarson, D. A. and Beyers, N. (2012) The Rate of Sputum Smear-Positive Tuberculosis after Treatment Default in a High-Burden Setting: A Retrospective Cohort Study, *PLoS ONE*, 7 (9). DOI:10.1371/JOURNAL.PONE.0045724.

Marx, F. M., Dunbar, R., Enarson, D. A., Williams, B. G., Warren, R. M., Van Der Spuy, G. D., Van Helden, P. D. and Beyers, N. (2014) The Temporal Dynamics of Relapse and Reinfection Tuberculosis After Successful Treatment: A Retrospective Cohort Study, *Clinical Infectious Diseases*, 58 (12), pp. 1676–1683. DOI:10.1093/cid/ciu186.

Naidoo, K. and Dookie, N. (2018) Insights into Recurrent Tuberculosis: Relapse Versus Reinfection and Related Risk Factors, *Tuberculosis*. DOI:10.5772/INTECHOPEN.73601.

Tomeny, E. M., Nightingale, R., Chinoko, B., Nikolaidis, G. F., Madan, J. J., Worrall, E., *et al.* (2022) TB morbidity estimates overlook the contribution of post-TB disability: evidence from urban Malawi, *BMJ Global Health*, 7 (5), pp. e007643. DOI:10.1136/BMJGH-2021-007643.

Wilkinson, T., Sculpher, M. J., Claxton, K., Revill, P., Briggs, A., Cairns, J. A., *et al.* (2016) The International Decision Support Initiative Reference Case for Economic Evaluation: An Aid to Thought, *Value in Health*, 19 (8), pp. 921–928. DOI:10.1016/j.jval.2016.04.015.

World Health Organization (2021) *Global tuberculosis report*. Geneva.

World Health Organization (2022) *WHO consolidated guidelines on tuberculosis*. Geneva.

Xiang, L., Pan, Y., Hou, S., Zhang, H., Sato, K. D., Li, Q., Wang, J. and Tang, S. (2016) The impact of the new cooperative medical scheme on financial burden of tuberculosis patients: evidence from six counties in China, *Infect Dis Poverty*, 5 (1), pp. 8. DOI:10.1186/s40249-015-0094-5.

Xiong, X., Zhang, Z., Ren, J., Zhang, J., Pan, X., Zhang, L., Gong, S. and Jin, S. (2018) Impact of universal medical insurance system on the accessibility of medical service supply and affordability of patients in China. DOI:10.1371/journal.pone.0193273.

Zhao, Q., Wang, L., Tao, T. and Xu, B. (2013) Impacts of the ‘transport subsidy initiative on poor TB patients’ in Rural China: a patient-cohort based longitudinal study in rural China, *PLoS ONE*, 8 (11), pp. e82503. DOI:10.1371/journal.pone.0082503.
